# Would You Buy Plant-Based Beef Patties? A Survey on Product Attribute Preference and Willingness to Pay among Consumers in Liaoning Province, China

**DOI:** 10.3390/nu14204393

**Published:** 2022-10-19

**Authors:** Mi Zhou, Boyao Guan, Li Huang

**Affiliations:** College of Economics and Management, Shenyang Agricultural University, Shenyang 110866, China

**Keywords:** choice experiment, product attribute preference, willingness to pay, MNL model

## Abstract

Carbon emissions from the production of beef account for the majority of carbon emissions from animal husbandry, and animal husbandry, in turn, is the main driver of global carbon emissions. At present, there are relatively few studies of consumer preferences for beef substitutes, especially in developing countries. However, plant-based meat is of great significance in solving the tense relationship between supply and demand, ensuring sustainable development, further improving food safety, and improving animal welfare. Based on survey data from 1146 consumers in Liaoning province, China, this paper adopts the choice experiment method to study consumer preferences and willingness to pay for four types of plant-based beef patty product attributes, traceability, safety certification, brand, and price, using the multivariate logit (MNL) model. The results show that consumers show greater preference and willingness to pay for plant-based beef patties with strong traceability, fully disclosed safety certification information, and mature production technology. Consumers generally have strong brand preferences, while there are some differences in preference for other product attributes. In addition, environmentally-focused consumers have a greater degree of preference for traceable products. This study explores the micro decision mechanism of the purchase of plant-based meat products in developing countries. The research conclusions of this paper have guiding significance for businesses engaged in plant-based meat production and processing because of the addition and use of attribute tags. This study also has reference significance for the regulatory standards of decision-making departments and government investment.

## 1. Introduction

By 2050, the global population is expected to reach 9 billion, leading to an increase of 73% in global demand for meat, such as poultry, pork, and beef [1]. Additionally, beef consumption in China is projected to increase by 119% between 2015 and 2050 [2]. Meat shortages may be a major sustainability challenge for China [3].

Industrial animal husbandry is a leading cause of environmental damage, biodiversity loss, and climate change [4,5,6,7]. A large amount of animal manure is produced during the livestock breeding process. In China, only 43% of manure is used on farmland, and a large portion is discarded [8]. This poor manure management results in significant greenhouse gas emissions, including carbon dioxide, nitrous oxide, and methane [9,10]. Cattle farming produces the most emissions, accounting for about 65% of livestock emissions [11]. In addition, high intake of red meat has many adverse effects on human health, including an increased risk of cancer and cardiovascular disease [12,13,14]. Eating meat also harms animals [15,16,17]. In order to alleviate a series of problems caused by meat, plant-based meat substitutes have gradually become more popular.

Existing research in this area has focused on consumer attitudes and acceptance of meat substitutes, such as plant-based meat or artificial meat. For example, Zhang et al. (2021) [18] focused on the research history of artificial meat, the current technological challenges, and their possible solutions, while analyzing consumer attitudes toward artificial meat. Pater et al. (2022) [19] examined the attitudes 8- to 10-year-old non-vegetarian Dutch children had towards plant-based meat analogues. Hinrichs et al. (2022) [20] expanded knowledge on the meat–masculinity link by examining whether negative attitudes toward plant-based eating help explain why these gender differences occur. Padilha et al. (2022) [21] provided new insight into consumers’ attitudes towards different protein products and the factors associated with the acceptance of lab-grown chicken and beef. They measured and compared 1078 Australian consumers’ beliefs, regarding conventionally raised meat (chicken and beef), plant-based meat alternatives, and lab-grown meat products across six attributes: health, safety, affordability, enjoyment, animal welfare, and environmental friendliness. In order to gain insights into the factors determining whether people eat meat alternatives, Marcus et al. (2022) [22] extended the theory of planned behavior (TPB) with three factors: animal welfare, environmental, and health concerns. Based on a quantitative online study conducted in Germany, they empirically tested the hypothesized model using structural equation modeling. Studies have also looked at consumer preferences for plant-based meat product attributes. For example, Asioli et al. (2021) [23] explored consumer preferences and willingness to pay for “in-vitro meat” from four aspects: production method, carbon trust label, antibiotic use, and price through choice experiments.

The existing studies provide important references for the selection of research methods and food product attributes in this paper. However, there are still questions left unaddressed. First, most of the current studies on plant meat focus on the acceptance of plant-based meat products or the development prospects of the plant-based meat alternative industry, with little investigation of the willingness to pay for plant-based meat products. Second, as important identifying attributes of products, product safety attributes and origin brand attributes are widely used to measure consumer preferences [24,25,26,27,28], while few studies have applied these two attributes to preferences for plant-based meat product attributes. Third, current studies on the influence of consumer characteristics on product preferences generally focus on gender, age, disposable income, and education [29,30,31] These studies do not discuss the influence of other personal characteristics on product attribute preferences. Finally, most articles discuss food safety legislation from a policy perspective, such as by addressing the European regulations, directives, and decisions regulating veterinary health and food safety [32,33,34,35]. Few articles discuss food safety legislation made by the decision-making departments and government from the perspective of consumers. However, the discussion of food safety legislation, from the perspective of consumers, also has some reference significance.

The present study was designed to help improve understanding of consumer preferences and their willingness to pay for plant-based beef patty product attributes. There have not been any studies that use choice experiments to verify the micro-determination mechanism of plant-based meat product purchases by consumers in developing countries. This study complements research on the acceptance of plant-based meat products in developing countries. Furthermore, this study investigates preference differences, based on different individual consumer characteristics. Given that plant-based meat products are environmentally friendly and low-carbon, this paper also focuses on the differences in attribute preferences of consumers focused on environmental issues. Finally, the research conclusions of this paper have guiding significance for businesses involved in plant-based meat production and processing, in terms of the addition and use of attribute tags. It also has reference significance for the regulatory standards of decision-making departments and government investment.

## 2. Research Design

### 2.1. Product Selection and Attribute Design

Plant-based beef patties are the focus of this study, and discrete preference choice experiment data is used for analysis. According to the 2021 China Statistical Yearbook, China’s per capita meat consumption showed a fluctuating trend from 2014 to 2020, but the per capita beef consumption has been showing an upward trend each year. Meanwhile, the “2021 China Plant Meat Industry Insights White Paper” pointed out that the cattle industry accounts for about 65% of total greenhouse gas emissions in the livestock sector. About 56.6 kg of carbon dioxide is emitted for every 1 kg of beef produced. Therefore, beef is facing problems, such as high market demand and negative impact on the environment, necessitating this research.

Before conducting the experiment, it is necessary to determine the specific product attributes of the plant-based beef patties. It was found that respondents paid more attention to the traceability, safety certification, brand, and price of beef patties than to their other attributes. Therefore, this paper sets these four attributes for the plant-based beef patties in the selection experiment.

Additionally, price is a major factor affecting willingness to pay for food [36]. Based on the results of a previous packaging survey on major domestic e-commerce platforms, this study determined that the average price of plant-based beef patties is 40 yuan/250 g, and two prices of 10 yuan/250 g and 25 yuan/250 g are set to account for the price elasticity of beef. See Table 1 for specific attributes.

### 2.2. Experimental Design

According to the attribute levels set in Table 1, a total of 81 attribute combinations can be designed (3 × 3 × 3 × 3 = 81). Since there are 85,320 ($C_{81}^{3}\;$= 85,320) selection sets of plant-based beef patties, consisting of 81 product combinations, consumers cannot answer all the selection sets. In this paper, five sets are randomly selected from the 85,320 choice sets, and each consumer completes the product selection in one set of choice sets. The probability of consumers choosing each set is the same.

Within each choice set, consumers were asked to make their own preference choices among three combinations of plant-based beef patty product attributes. In order to make the experimental environment closer to the real market, the option “None of the above” is included in each choice set, which means that none of the three attribute combinations in the selection set increase the utility of consumers. Consumers will choose to be content with the status quo and will not change their current consumption behavior. When making the choice, consumers will be informed that there is no difference in any of the attributes, except those involved in the selection experiment, and that each plant-based beef patty weighs 250 g. Table 2 is an example of a choice set.

### 2.3. Data and Descriptive Statistics

This paper uses Questionnaire.com (https://www.wenjuan.com/ accessed on 13 October 2022) to conduct online research (Questionnaire.com is a large, free online survey platform in China, which allows for the creation, release, management, and collection of questionnaires. The questionnaire network platform has a rich and complete sample database, and sample groups can be selected from different regions, ages, income levels, and other characteristics for investigation. This paper mainly looks at the living area of the respondents). A pre-survey was conducted in November 2021, and a formal survey was carried out from December 2021 to February 2022. Questionnaires for the selection experiment include the consumer personal characteristics and selection experiments of different combinations of plant-based beef patty product attributes.

The survey covers all 14 prefecture-level cities in Liaoning Province, China. Data from a total of 1146 consumers were obtained after deleting invalid questionnaires with missing values and logical inconsistencies that affected the experiment conclusions. Male consumers accounted for 31% of the sample, which means that women usually play an important role in household food shopping, as mentioned in the study by Zhang et al. (2012) [37]. The variable definition and descriptive statistics are presented in Table 3.

## 3. Analytic Approach

### 3.1. Theoretical Framework

Based on the behavioral analysis framework of stochastic utility theory, the choice experiment method provides decision makers with choice sets composed of different attribute states [38]. According to the utility maximization assumption, the maximum utility obtained by the decision maker in a choice set is:

(1) maxU=U(γx|p,E)

where x is the product, p is the corresponding product price, and E is the budget revenue; γ∈[0,1] represents function the respondent expects the product to have, where γ = 0 indicates that the expected function is not realized, and γ = 1 indicates that the expected function is realized. The utility obtained by a consumer consuming product x is proportional to the expected function the consumer expects from the product. The more the expected function is achieved, the greater the utility, and vice versa. At the same time, the demand for a product moves in the same direction as the income level of the consumer.

Functional expectations of products are related to consumption motivations for those products. The nutritional, health, and low-carbon attributes of plant-based meat may become important factors driving consumers to purchase and eat plant-based beef patties.

### 3.2. Analytical Model

This paper adopts the multivariate logit model (MNL) to analyze the plant-based beef patty product attributes that affect consumer preferences. Discrete choice analysis usually assumes a utility function:

(2) $$U_{\mathrm{ij}}=V_{\mathrm{ij}}+\varepsilon_{\mathrm{ij}}=\beta_{0}+\beta_{p}P_{\mathrm{ij}}+\beta_{x}X_{\mathrm{ij}}+\varepsilon_{\mathrm{ij}}$$

where $U_{ij}$ is the indirect utility obtained by individual i choosing product combination j; $V_{ij}$ is the deterministic part of utility; $\varepsilon_{ij}$ is the error term; $\beta_{0}$ is the constant term; $P_{ij}$ represents the price attribute of the product, when individual i chooses product combination j; $\beta_{p}$ is the correlation coefficient of $P_{ij}$, which is the marginal utility of the price attribute; $X_{ij}$ represents non-price attributes. In this paper, non-price attributes refer to the traceability, safety certification, and brand attributes of plant-based beef patties. $\beta_{x}$ is the correlation coefficient of $X_{ij}$, which is the marginal utility of other product attributes. The probability that consumer i chooses product combination j in the combination set can be expressed as:

(3) $$P_{\mathrm{ij}}=\frac{\exp\left(V_{\mathrm{ij}}\right)}{\sum_{j}\exp\left(V_{\mathrm{ij}}\right)}$$

### 3.3. Calculation of Willingness to Pay

Consumer willingness to pay for certain attributes of a product refers to the highest premium that consumers are willing to pay to keep their own utility unchanged when other attributes remain unchanged. Consumer willingness to pay for the change of a certain attribute can be expressed as:

(4) $$\beta_{0}+\beta_{p}P_{\mathrm{ij}}+\beta_{x}X_{\mathrm{ij}0}+\overset{k}{\underset{k=2}{\sum }}\beta_{\mathrm{xk}}X_{\mathrm{ijk}}+\varepsilon_{\mathrm{ij}}\;=\beta_{0}+\beta_{p}(P_{\mathrm{ij}}+{\mathrm{WTP}}_{\mathrm{ij}1})+\beta_{x}X_{\mathrm{ij}1}+\overset{k}{\underset{k=2}{\sum }}\beta_{\mathrm{xk}}X_{\mathrm{ijk}}+\varepsilon_{\mathrm{ij}}$$

$X_{\mathrm{ij}0}$ represents an attribute level before the change; $X_{\mathrm{ij}1}$ represents the attribute level after the change; $X_{\mathrm{ijk}}$ represents other unchanged product attribute levels; $\beta_{\mathrm{xk}}$ is the correlation coefficient of $X_{\mathrm{ijk}}$, which is the marginal utility; ${\mathrm{WTP}}_{\mathrm{ij}1}$ is the willingness of consumers to pay for the changed attributes.

According to Formula (4), this can be written as:

(5) $${\mathrm{WTP}}_{\mathrm{ij}1}=-\frac{\beta_{x}}{\beta_{p}}$$

## 4. Results

### 4.1. Consumer Preference for Each Product Attribute of Plant-Based Beef Patties

Compared with effect coding, the welfare estimation results of dummy coding are easier to interpret and less prone to welfare estimation errors [39]. In this paper, dummy coding is used to assign values to the product attributes of plant-based beef patties. The first level of each attribute in Table 1 is taken as the basic level, and the MNL model is used to analyze consumer product preferences for plant-based beef patties.

As shown in Table 4, the coefficient of the price attribute is significantly negative at the 1% level, indicating that consumer preference for plant-based beef patty products declines with increasing product prices, which is in line with previous expectations.

Traceability to processing and production both have a significant positive effect on consumer preferences, and the results are statistically significant at the 1% level. This indicates that, with all conditions being equal, consumers prefer products with traceable attributes, and that traceability to production has a greater impact on consumer utility than traceability to processing. With more product traceability, consumers can obtain more information about the production and processing of the products, leading to greater preference for these products. This is similar to the conclusion of Ortega et al. (2011) [40], i.e., that consumer willingness to pay for product traceability labels is significantly.

Both coefficients of the origin safety certification and the origin and place of sale safety certification attributes are significant at the 1% level. This suggests that, all things being equal, consumers prefer origin safety certification attributes and origin and place of sale safety certification attributes to sale place safety certification. This is consistent with the previous conclusion of Qiao et al. (2021) [41], i.e., that consumers pay a higher premium for pork that contains origin labels. In addition, consumers prefer origin and place of sale safety certifications to origin safety certifications, which indicates that fully guaranteeing the quality and safety of products significantly increases consumer preference and willingness to pay.

In terms of brands, national brands have a significant negative effect on consumer preference, relative to local brands (*p* < 0.01). In other words, consumers prefer local brands to national brands, which is consistent with findings by Shang et al. (2014) [42], which show that consumers have a greater preference for locally produced honey. Compared with local brands, foreign brands have a significant positive effect on consumer preference (*p* < 0.01), which indicates that, among brand attributes, consumers have the greatest preference for foreign brands. This may be because the domestic plant-based meat technology is developing slower than foreign industries, and the products in the market with the best taste and appearance are foreign brands.

### 4.2. Consumer Willingness to Pay for Each Product Attribute of Plant-Based Beef Patties

Table 5 shows consumer willingness to pay for each product attribute of plant-based beef patties, and the results are calculated based on the marginal utility of the product attributes in Table 4.

In terms of traceability, consumer willingness to pay for products traceable to processing is 18.53 yuan/250 g, and for the those traceable to production, it is 55.47 yuan/250 g, indicating that consumer willingness to pay for the attribute of product traceability increases with the level of traceability. In terms of safety certification, consumer willingness to pay for origin safety certification is 44.05 yuan/250 g, and for origin and place of sale, safety certification it is 119.16 yuan/250 g, reflecting that consumers are more willing to pay for products whose safety and quality are fully guaranteed. For brand attributes, consumer willingness to pay for domestic brands is −71.42 yuan/250 g, while that for foreign brands is 34.26 yuan/250 g. Therefore, consumer willingness to pay for foreign brands far exceeds that of domestic brands.

### 4.3. Influence of Individual Characteristics on Consumer Preferences for Plant-Based Beef Patties

To further compare and investigate the heterogeneity of preference for the attributes of plant-based beef patties among consumers with different personal characteristics, the interaction terms of gender, age, and cognitions of green consumption were constructed. Table 6 shows the coefficient and significance of the interaction terms between product attributes and individual characteristics, and the t-statistics of the interaction terms are shown in parentheses.

First, women have a greater preference for the attributes of traceable to production, origin and place of sale safety certification, and foreign brand, while men prefer attributes of traceable to processing and domestic brands. Second, young consumers have a greater preference for foreign brand attributes. This is consistent with the conclusion of Hossain et al. (2021) [29], i.e., that young people are more willing to buy high-quality certified food than the elderly, and the elderly are generally reluctant to change their food consumption behavior and pay for new food attributes. Finally, self-rated green consumers have a greater preference for attributes that can be traceable to production.

## 5. Conclusions and Policy Recommendations

Using survey data from 1146 consumers in Liaoning Province, China, this paper adopts the method of choice experiment to analyze consumer preference and willingness to pay for the attributes of plant-based beef patties and explores the differences in consumer preferences for plant-based beef patties across different individual characteristics. This paper found that consumers showed high preference and willingness to pay for plant-based beef patties with strong traceability, fully disclosed safety certification information, and mature production technology. Although consumers with different characteristics have differences in their preferences for plant-based beef patty attributes, the empirical results show that consumers generally pay more attention to brands and prefer foreign brands to local and national brands.

These results have policy implications. Firstly, decision-making departments should consider issuing more relevant industry standards to regulate the safety of plant-based meat products to enhance consumer trust. Secondly, the government should increase investment in the plant-based meat production industry to enhance the competitiveness of domestic brands. As an alternative to traditional meat, plant-based meat can alleviate a series of problems brought about by traditional meat products. However, there is still a big gap in production and processing technology in China, compared with other countries. The Chinese government should encourage domestic plant-based meat production and processing enterprises to carry out technological innovation, so as to achieve product upgrading as soon as possible and enhance the competitiveness of domestic plant-based meat products.

Although this paper has made some contributions in verifying the micro-decision mechanism for consumers in developing countries to purchase plant-based meat products and analyzing the differences in the product attribute preferences of consumers who pay attention to environmental issues, there are still some limitations. For example, animal welfare may also be an important factor affecting consumer behavior. One limitation of this article is that animal welfare was not considered as an influencing factor affecting willingness to pay for plant-based beef patty products.

## Figures and Tables

**Table 1:** Attributes and levels used in choice experiment.

Attribute	Levels
Traceability	Not traceable
	Traceable to processing
	Traceable to production
Safety Certification	Sales place safety certification
	Origin safety certification
	Origin and place of sale safety certification
Brand	Local brand
	Domestic brand
	Foreign brand
Price	10 yuan/250 g
	25 yuan/250 g
	40 yuan/250 g

**Table 2:** Example of choice sets in the choice experiment.

Attributes	Product 1	Product 2	Product 3	
Safety Certification	Sales place safety certification	Sales place safety certification	Origin and place of sale safety certification	None of the above
Traceability	Not traceable	Traceable to production	Traceable to processing	
Brand	Local brand	Foreign brand	Domestic brand	
Price	10 yuan/250 g	25 yuan/250 g	40 yuan/250 g	

**Table 3:** Summary statistics.

Variables	Variable Description	Obs.	Mean	S.D.	Min.	Max.
Gender	1 = male; 0 = female	1146	0.31	0.46	0	1
Age	1 = 20 years old and below, 2 = 21–30 years old, 3 = 31–40 years old, 4 = 41–50 years old, 5 = 51 years old and above	1146	2.83	0.88	1	5
Place of residence	1 = rural area, 2 = township, 3 = county town, 4 = prefecture-level city, 5 = provincial capital city	1146	4.25	0.89	1	5
Hukou type	1= agricultural, 0= non-agricultural	1146	0.26	0.44	0	1
Migrant worker	1= yes; 0= no	296	0.43	0.50	0	1
Education	1 = primary school and below, 2 = junior high school, 3 = technical secondary school, 4 = senior high school, 5= junior college, 6 = undergraduate, 7 = Master and above	1146	5.46	0.95	1	7
Family size	Number	1146	3.16	1.03	1	6
Disposable income	1 = 1000 or less, 2 = 1000–2000, 3 = 2000–3000, 4 = 3000–4000, 5 = 4000–5000, 6= more than 5000	1146	4.49	1.59	1	6
Green Consumer	1= yes; 0= no	1146	0.46	0.50	0	1

Note: Hukou type is divided into non-agricultural household registration and agricultural household registration. Consumers whose Hukou type is agricultural household registration and who have been engaged in non-agricultural work outside the county for more than six months are defined as migrant workers. Consumers who are willing to pay a higher price for environmentally-friendly goods are defined as green consumers. The consumers stated whether they considered themselves green consumers.

**Table 4:** Consumer preference for each product attribute of plant-based beef patties.

Attribute	Levels	Coefficient	*t*-Value	95%CI
Traceability	Traceable to processing	0.352 ***	3.204	[0.788, 1.319]
	Traceable to production	1.054 ***	7.775	[0.137, 0.567]
Safety Certification	Origin safety certification	0.837 ***	10.070	[0.674, 1.000]
	Origin and place of sale safety certification	2.264 ***	6.732	[1.605, 2.923]
Brand	Foreign brand	0.651 ***	6.115	[−0.011, 0.536]
	Domestic brand	−1.357 ***	−6.700	[−1.754, −0.960]
Price		−0.019 ***	−3.748	[−0.030, −0.009]
Sample size	4584			

Note: Each participant faces four choices when making product selection, and the sample size is 4584 (1146 × 4 = 4584), the same as below. Significance: *** *p* < 0.01.

**Table 5:** Consumer willingness to pay for each product attribute of plant-based beef patties (yuan/ 250 g).

Attribute	Levels	WTP
Traceability	Traceable to processing	18.53
	Traceable to production	55.47
Safety Certification	Origin safety certification	44.05
	Origin and place of sale safety certification	119.16
Brand	Foreign brand	34.26
	Domestic brand	−71.42
Sample size	4584	

Note: WTP is the willingness of consumers to pay for the changed attributes.

**Table 6:** Influence of individual characteristics on consumer preferences for plant-based beef patties.

Variables	Gender	Age	Self-Rated Green Consumers
Traceable to processing	0.531 ** (2.359)	0.153 (1.251)	0.248 (1.210)
Traceable to production	−0.416 ** (−2.208)	−0.164 (−1.616)	0.431 *** (2.580)
Origin safety certification	−0.0002 (−0.001)	−0.107 (−1.138)	−0.160 (−1.027)
Origin and place of sale safety certification	−2.213 *** (−4.527)	−0.306 (−1.098)	0.701 (1.506)
Foreign brand	−0.571 *** (−3.010)	−0.186 * (−1.818)	−0.115 (−0.674)
Domestic brand	1.642 *** (4.199)	0.357 (1.555)	−0.282 (−0.733)

Note: Significance: * *p* < 0.10, ** *p* < 0.05, *** *p* < 0.01.

## Data Availability

The data presented in this study are available on request from the corresponding author.
